# Munchausen Syndrome by Proxy in Singapore

**DOI:** 10.1192/bjo.2025.10742

**Published:** 2025-06-20

**Authors:** Derrick Yeo, Shannon Lee

**Affiliations:** 1 Institute of Mental Health, Singapore, Singapore; 2 Ministry of Health Holdings, Singapore, Singapore

## Abstract

**Aims:** Munchausen syndrome by proxy, or “factitious disorder imposed on another” as per DSM–V criteria, is characterised by the falsification of signs or symptoms, or induction of injury or disease, in another individual. Despite its initial description over 60 years ago, the literature on its epidemiology, management, and prognosis remains limited, with most insights derived from isolated case reports.

**Methods:** We report a case of a 27-year-old woman charged with attempted murder after injecting her 7-year-old son with insulin multiple times. The patient’s actions were driven by a history of severe childhood trauma, including sexual abuse by her father and brother, which contributed to her distorted perceptions of her son’s behaviour. She falsely presented symptoms to healthcare providers, altered diagnostic tests, and fabricated medical histories, resulting in extensive and unnecessary investigations for the child on top of complications from being injected by insulin. Psychiatric evaluation diagnosed her with major depressive disorder, post-traumatic stress disorder, antisocial personality traits, and Munchausen syndrome by proxy. Despite being aware of the harm caused by her actions, the patient’s judgement was significantly impaired due to her mental illnesses. Treatment included antidepressants and psychotherapy, with partial improvement observed.

**Results:** This case illustrates the significant risks posed by Munchausen syndrome by proxy to victims and the complexities involved in its diagnosis and management. Early identification requires a high index of suspicion and meticulous investigation by multidisciplinary teams. Video surveillance and psychiatric evaluations are crucial tools in confirming such cases. Long-term management often necessitates pharmacological treatment and tailored psychotherapy for the perpetrator, alongside safeguarding measures for the victim.

**Conclusion:** Munchausen syndrome by proxy remains a challenging diagnosis requiring vigilance and interdisciplinary collaboration. This case underscores the importance of early recognition to prevent harm to victims and highlights the need for systematic research to explore common patterns and effective interventions in this rare condition.

